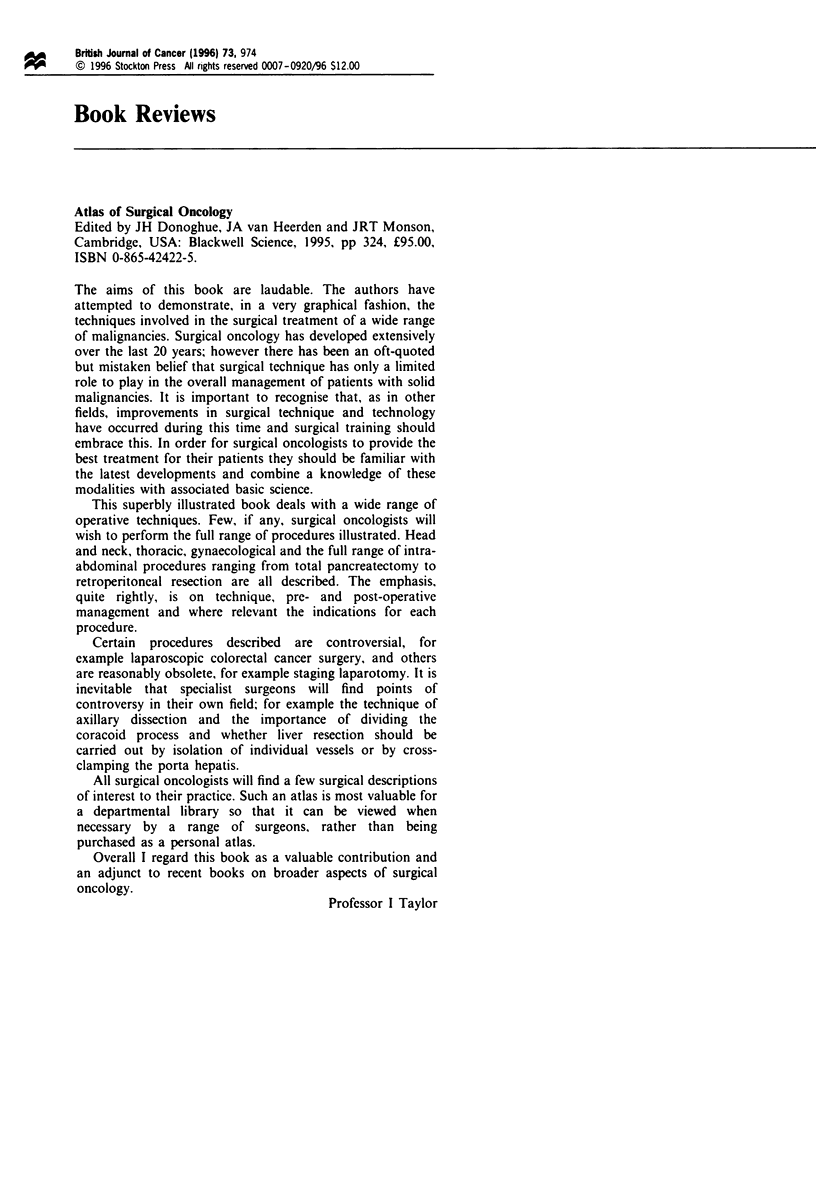# Atlas of Surgical Oncology

**Published:** 1996-04

**Authors:** I Taylor


British Journal of Cancer (1996) 73, 974
© 1996 Stockton Press All rights reserved 0007-0920/96 $12.00

### Book Reviews

**Atlas of Surgical Oncology**
Edited by JH Donoghue, JA van Heerden and JRT Monson, Cambridge, USA: Blackwell Science, 1995, pp 324, £95.00, ISBN 0-865-42422-5.

The aims of this book are laudable. The authors have attempted to demonstrate, in a very graphical fashion, the techniques involved in the surgical treatment of a wide range of malignancies. Surgical oncology has developed extensively over the last 20 years; however there has been an oft-quoted but mistaken belief that surgical technique has only a limited role to play in the overall management of patients with solid malignancies. It is important to recognise that, as in other fields, improvements in surgical technique and technology have occurred during this time and surgical training should embrace this. In order for surgical oncologists to provide the best treatment for their patients they should be familiar with the latest developments and combine a knowledge of these modalities with associated basic science.

This superbly illustrated book deals with a wide range of operative techniques. Few, if any, surgical oncologists will wish to perform the full range of procedures illustrated. Head and neck, thoracic, gynaecological and the full range of intra-abdominal procedures ranging from total pancreatectomy to retroperitoneal resection are all described. The emphasis, quite rightly, is on technique, pre- and post-operative management and where relevant the indications for each procedure.

Certain procedures described are controversial, for example laparoscopic colorectal cancer surgery, and others are reasonably obsolete, for example staging laparotomy. It is inevitable that specialist surgeons will find points of controversy in their own field; for example the technique of axillary dissection and the importance of dividing the coracoid process and whether liver resection should be carried out by isolation of individual vessels or by cross-clamping the porta hepatis.

All surgical oncologists will find a few surgical descriptions of interest to their practice. Such an atlas is most valuable for a departmental library so that it can be viewed when necessary by a range of surgeons, rather than being purchased as a personal atlas.

Overall I regard this book as a valuable contribution and an adjunct to recent books on broader aspects of surgical oncology.

Professor I Taylor